# Effect of a Culturally Adapted Exercise Program on the Anthropometrics and Body Composition of Postmenopausal Women With Excess Weight Gain: A Randomized Controlled Trial

**DOI:** 10.1002/osp4.70038

**Published:** 2025-01-07

**Authors:** Isaac Mensah Bonsu, Corlia Brandt, Adedayo Tunde Ajidahun, Hellen Myezwa

**Affiliations:** ^1^ Department of Physiotherapy School of Therapeutic Sciences University of the Witwatersrand Johannesburg South Africa; ^2^ College of Health Sciences, Faculty of Allied Health Sciences Department of Physiotherapy and Sports Science Kwame Nkrumah University of Science and Technology Kumasi Ghana

**Keywords:** anthropometrics, body composition aging, cultural, exercise program, postmenopausal women

## Abstract

**Background:**

Physical activity (PA) is recommended as a component of weight management for the prevention of weight gain and weight regain after weight loss. Yet, no study has adapted culturally appropriate PA for postmenopausal women's health.

**Aims:**

The study aimed to investigate the effect of a developed culturally appropriate exercise program for Ghanaian postmenopausal women with excess weight gain on the anthropometrics and body composition.

**Material and methods:**

A single‐blind randomized controlled trial in which participants randomly received a culturally‐induced exercise program. A total of 226 Ghanaian postmenopausal women were randomized into exercise and control groups for 12 weeks. Anthropometrics (body mass index [BMI], waist‐to‐hip ratio [WHR], waist‐to‐height ratio [WHtR] waist circumference [WC], hip circumference [HC], and weight) and body composition (body fat, visceral fat, muscle mass) were determined pre‐and post‐intervention.

**Results:**

Average of 58.70 ± 6.38 years (*p* > 0.05) with more than half (52.1%) above 58 years. Except for WHR, there were statistically significant differences in weight, BMI, WHtR, visceral fat, and total body fat in the exercise and control groups. Muscle mass increased significantly (+0.21 kg), whereas HC (−2.46 cm) and WC (−1.39 cm) decreased significantly compared with the control group. Within the exercise group, when stratified by BMI, there were higher reductions in BMI (1.01 kg/m^2^ vs. 0.46 kg/m^2^), WC (2.18 cm vs. 0.22 cm), body weight (2.12 kg vs. 1.17 kg) and body fat (1.84% vs. 1.6%) in women with obesity compared with women with overweight.

**Conclusion:**

The promotion of Indigenous physical activity in postmenopausal women is beneficial. This has implications for health professionals who prescribe physical activity in postmenopausal women's treatment plans.

**Trial Registration:**

PACTR202301779437544

## Graphical abstract

A 12‐week culturally adapted exercise program combining aerobic activity and resistance exercises positively influenced body composition and anthropometrics of postmenopausal women.

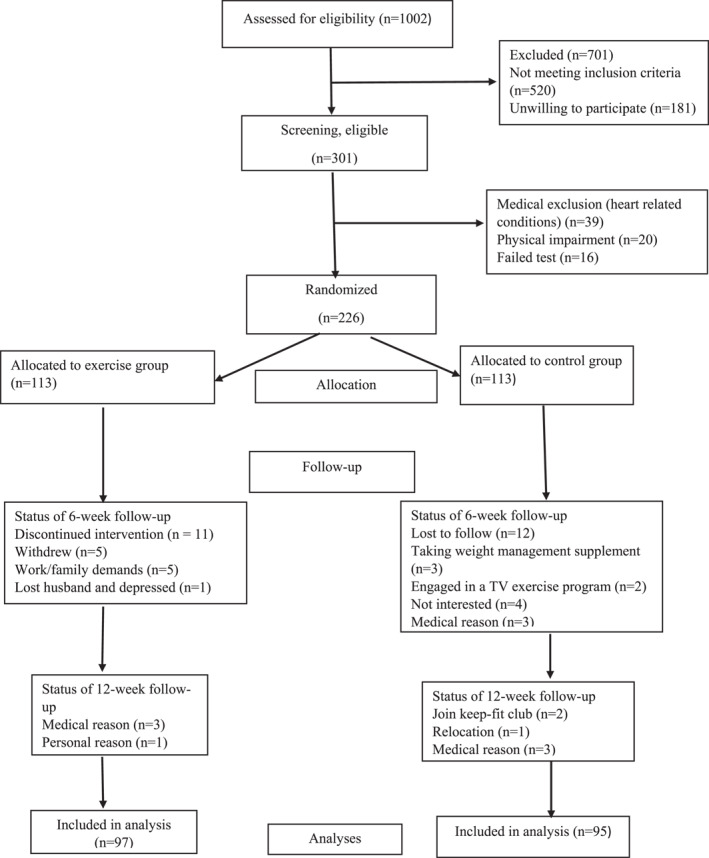

## 1 Introduction

Menopause is generally associated with several changes that can worsen women's health‐related fitness, particularly when combined with a sedentary lifestyle as well as weight gain [1]. To date, research has demonstrated that postmenopausal women frequently present with an increased prevalence of risk factors for cardiovascular disease (CVD) [1, 2, 3]. Most often associated with weight gain [4], postmenopausal women gradually lose lean body mass and cardiovascular health [5], and there is an increase in visceral fat and total body fat [6], which contribute considerably to the rise in morbidity and death rates [7]. Additionally, the physical activity level in women decreases with aging, which also results in adverse alterations to their body composition [8].

Evidence‐based recommendations on the management of weight gain encourage the use of waist circumference as a measurement of abdominal obesity for determining the relative risk of disease in a person with excess weight gain [9]. Therefore, interventions that specifically aim to reduce abdominal fat may be more successful in lowering the CVD risk associated with excess weight in postmenopausal women [10].

A systematic review and meta‐analysis investigating the impact of exercise training on body composition in postmenopausal women found that exercise significantly increased muscle mass/volume, muscle and fiber cross‐sectional area, and fat‐free mass while reducing fat mass, body fat percentage, waist circumference, and visceral fat [1]. Subgroup analysis within the same review revealed that aerobic and combined training was more effective in reducing fat mass, whereas resistance and combined training showed greater benefits in improving muscle mass outcomes [1]. The effect of exercise on body composition has been validated in subsequent studies that investigated the benefits of aerobic and muscle strength training separately and in some cases in combination with a weight loss intervention in postmenopausal women [11, 12, 13, 14, 15]. These studies generally demonstrated that aerobic exercise decreases body weight and total fat, whereas muscle strength training increases lean body mass. Based on these findings and the recommendations of the American College of Sports Medicine, aerobic and resistance exercises are estimated to have the most beneficial effects on body composition [16].

The positive effects of an exercise program on total body fat, body weight, and lean mass are undisputed [11, 12, 13, 14, 15]; however, no study has investigated the effect of culturally appropriate exercise programs on postmenopausal women's body composition and anthropometrics. This study was part of a bigger project to understand the cultural perspective of physical activity on women's health. As a result, this study aimed to examine the effect of a culturally adapted exercise program in the Ghanaian context on the anthropometrics and body composition of postmenopausal women with excess weight gain.

## 2 Method

### 2.1 Study Design

This study was a single‐blind randomized controlled trial conducted at the Bono‐East regional capital, Techiman, over 3 months (from June to August 2022). This study investigated the effect of an exercise program developed in a Ghanaian context on anthropometrics and body composition of postmenopausal women with excess weight gain.

### 2.2 Selection/Recruitment Process

Participants within the Bono‐East regional capital, Techiman, were recruited through local advertisements in the markets, churches, mosques, and community. An announcement was made in the women's association, churches, and mosque meetings 1 week before the recruitment, while various queen mothers in the markets announced the study to the women through information centers 3 days before recruitment. Postmenopausal women were enrolled based on the following criteria: (1) BMI ≥ 25 kg/m^2^, (2) age 48–70 years, (3) postmenopausal status (no menses for 0.1 year), (4) not engaged in weight management programs and sedentary lifestyle (15 min exercise 2 times/week and not adherent to the WHO physical activity recommendation). Participants were excluded if they had a medical condition that might be adversely affected by increasing their physical activity, as determined by the physical activity readiness questionnaire (PAR‐Q) [17]. Teaching Assistants from the Department of Physiotherapy and Sports Science at Kwame Nkrumah University of Science and Technology were specifically trained by the principal investigator (IMB) as research assistants. Three of these researchers carried out the recruitment process.

**FIGURE 1:**
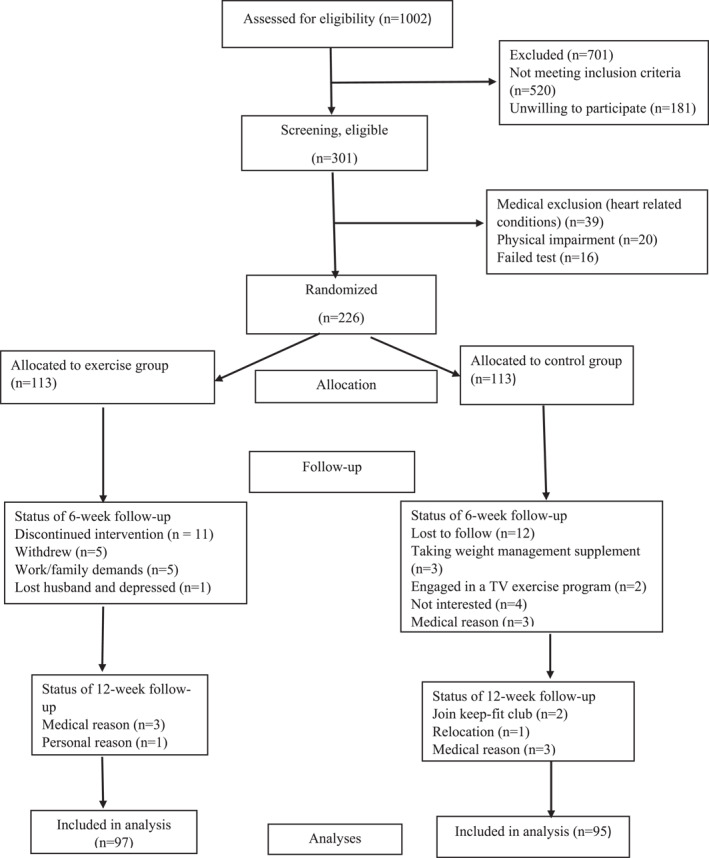
Sample selection, allocation and follow‐up.

A total of 1002 women were initially screened from ten (10) different study sites (churches, women's organizations, markets, mosques, and communities) (Figure 1). Of those women, 301 were medically screened and underwent an exercise test (step tests) to determine their aerobic fitness at each study site. Out of the population of 301, 226 were enrolled in the study and randomized into the control and exercise.

### 2.3 Sample Size Calculation

All Ghanaian postmenopausal women with excess weight gain constituted a sample frame. With a 20% dropout rate, a sample size of 270 was determined.

### 2.4 Randomization and Blinding

On each of the sites, participants were randomized into exercise and control groups using blocks of 4, 8, and 12 chosen with equal probability. A total of 226 were randomly assigned to either the control group (*n* = 113) or the exercise group (*n* = 113). Allocation concealment was maintained in this study until written consent was obtained along with demographics and the completion of baseline measures. Following baseline measurement, a researcher opened a sequentially numbered opaque envelope containing the allocated group (exercise or control) and showed the allocation to the participant. The researcher assessing participants at 6‐ and 12‐week follow‐up visits was blind to group allocation, and participants were requested not to discuss group allocation. The principal investigator (IMB) was blinded to group allocation while assessing the outcome measures and plotting and analyzing the data. Participants agreed to maintain their baseline level of physical activity if not assigned to the intervention for the period of our study. All participants accepted the randomization results after a detailed information session. The control group did not receive any specified program; however, measuring their vital signs (blood pressure) and other anthropometrics were enough incentives and motivation for them. Women in both groups were asked to maintain their lifestyle (usual diet) and after 12 weeks, post‐intervention data were collected. Consort guidelines for reporting are shown in Figure 1 [18].

### 2.5 Exercise Program

The intervention was developed by a consensus study in a local context with cultural adaptation. The home‐based exercise program included Ghanaian indigenous physical activity (Ampe) that involves clapping while singing and jumping vertically. “Ampe” is an aerobic recreational activity that incorporates good physical workouts, critical thinking, and social bonding with intermittent active rest. Typically, two or more people, identified as “Ohyiwa and Opare,” are involved in “ampe” performance. “Ohyiwa” scores a point when her left leg meets the right leg or the right leg meets the left leg of “Opare” and vice versa. To play “ampe,” an individual manipulates the legs and alternately places one leg forward. Although “ampe” can be played competitively at low, moderate, or high intensity among multiple groups, it is primarily utilized as a leisure game or pastime that can be done alone.

Additionally, the exercise program had six (6) different resistance exercises, such as squat to chair, wall push‐ups, and exercises with water bottles. The details of the exercise dosage were explained, printed, and given to the participants in the exercise group. A trained female exercise instructor based in the community provided telephonic support (an average of three calls, each lasting 8 min) over the 12 weeks, encouraging and motivating them to overcome personal barriers to exercise. The women played the Ampe exercise in 3–4 sets, 3–4 times/week with 10–15 repetitions for a maximum of 10–15 min. The resistance exercises involved 4 sets, 3–5 times/week of 10 repetitions for 5–10 min. A high degree of consensus among both international and local experts was reached on the component of the exercise program (unpublished data).

### 2.6 Outcome Measurements

At baseline and after 12 weeks of intervention, participants' anthropometrics and body composition were obtained. Height was determined by a stadiometer (Ghaziabad, Scorpia India Medicare Pvt. Ltd. | ID: 4076377262) and an Omron Body Composition Monitor with Scale (Omron BF‐511T—rozbaleno) was used to measure weight, skeletal muscle mass, visceral fat area body fat percentage as well as BMI while the participants wore light clothes and no shoes. With the participant in a standing position (with feet together), an inelastic tape was used to measure waist and hip circumference (to the nearest 0.1 cm) as the smallest girth around the waist and the region of maximum girth around the buttocks—(the trochanteric region) for hip circumference [19]. The waist‐hip ratio (WHR) and waist‐to‐height ratio (WHtR) were determined. All these parameters were measured pre‐ and post‐intervention.

### 2.7 Statistical Analyses

Baseline characteristics were reported as means ± SD or as percentages and frequencies of the study groups. An independent *t*‐test was used to compare the baseline anthropometric and body composition measures between the intervention and control groups. Additionally, we performed stratified analyses to investigate variations in body composition and anthropometric changes within the exercise group (women with overweight vs. women with obesity) classified by baseline age (48–58 and 58+) using a repeated‐measures linear mixed model. Statistical analyses were performed using SPSS software version 26.

## 3 Results

The socio‐demographic characteristics of the 192 participants randomized to the exercise trial are shown in Table 1. Post‐menopausal women were on average 58.70 ± 6.38 years old with more than half (*n* = 100, 52.1%) above 58 years. Most of the participants (*n* = 104, 54.2%) had completed junior or senior high school. More than two‐thirds of them were Akans (*n* = 138, 71.9%), employed (*n* = 153, 79.7%), Christians (*n* = 154, 80.6%), and traders (*n* = 132, 68.8%) by profession. Almost three‐fifths (57.3%, *n* = 110) were divorced or married, respectively, whereas 24.0% (*n* = 46) were widowed (Table 1). Except for educational level (*p* = 0.037), there were no statistically significant differences in any demographic characteristic between the study groups at baseline (all *p* > 0.05).

**TABLE 1:** Socio‐demographic characteristics of the trial participants.

Characteristics	Total (*N* = 192)	Control (*N* = 95)	Exercise (*N* = 97)	*p*‐value
Age (years)	58.70 ± 6.38	58.83 ± 6.63	58.58 ± 6.15	0.783
Age group (years)				0.669
48–58	92 (47.9)	47 (49.5)	45 (46.4)	
58+	100 (52.1)	48 (50.5)	52 (53.6)	
Religion				0.307
Christians	154 (80.6)	73 (77.7)	81 (83.5)	
Muslims	37 (19.4)	21 (22.3)	16 (16.5)	
Marital status				0.306
Single	17 (8.9)	8 (8.4)	9 (9.3)	
Married	110 (57.3)	50 (52.6)	60 (61.9)	
Divorced	110 (57.3)	13 (13.7)	6 (6.2)	
Widowed	46 (24.0)	24 (25.3)	22 (22.7)	
Ethnicity				0.529
Akan	138 (71.9)	65 (68.4)	73 (75.3)	
Ga or Ewe	15 (7.8)	9 (9.5)	6 (6.2)	
Northerner	39 (20.3)	21 (22.1)	18 (18.6)	
Educational level				0.037
None	35 (18.2)	21 (22.1)	14 (14.4)	
Primary	31 (16.1)	21 (22.1)	10 (10.3)	
Junior or High	104 (54.2)	44 (46.3)	60 (61.9)	
Tertiary	22 (11.5)	9 (9.5)	13 (13.4)	
Occupation type				0.683
Trader	132 (68.8)	64 (67.4)	68 (70.1)	
Others	60 (31.3)	31 (32.6)	29 (29.9)	
Employment status				0.184
Unemployed	39 (20.3)	23 (24.2)	16 (16.5)	
Employed	153 (79.7)	72 (75.8)	81 (83.5)	

### 3.1 Baseline Anthropometric and Body Composition Measures of the Trial Participants

The baseline anthropometric and body composition measures of the trial participants are presented in Table 2. Except for muscle mass with a higher mean in the control than in the exercise group (25.45 ± 3.62 versus 24.68 ± 2.47), all other anthropometrics and body composition measures at pre‐test (baseline) were higher in the exercise group compared to the control group (all *p* > 0.05).

**TABLE 2:** Baseline anthropometric and body composition measures of the trial participants (Ghana 2022).

Variables	Total (*N* = 192)	Control (*N* = 95)	Exercise (*N* = 97)	*p*‐value^a^
Weight (kg)	77.81 ± 12.01	76.67 ± 10.80	78.91 ± 13.07	0.197
Height (cm)	157.48 ± 7.17	157.24 ± 6.52	157.71 ± 7.77	0.651
BMI (kg/m^2^)	31.43 ± 4.43	31.19 ± 4.56	31.67 ± 4.31	0.449
Muscle mass (kg)	25.06 ± 3.11	25.45 ± 3.62	24.68 ± 2.47	0.090
Body fat (%)	42.65 ± 5.47	42.09 ± 5.39	43.19 ± 5.52	0.164
Visceral fat	11.02 ± 1.93	10.95 ± 2.04	11.08 ± 1.82	0.628
WC (cm)	101.21 ± 9.44	100.03 ± 9.22	102.36 ± 9.57	0.086
HC (cm)	112.22 ± 9.66	111.57 ± 8.76	112.87 ± 10.47	0.353
WHR	0.90 ± 0.06	0.90 ± 0.07	0.91 ± 0.06	0.246
WHtR	0.64 ± 0.06	0.64 ± 0.06	0.65 ± 0.06	0.143

^a^ *p* < 0.05 versus baseline for that group. For within‐group comparisons between post‐test and pre‐test values, a repeated‐measures linear mixed model was used.

### 3.2 Changes From Baseline for Anthropometric and Body Composition Measures With the Intervention of the Exercise Trial

Anthropometric and body composition changes were evaluated at baseline and twelve weeks and are presented in Table 3.

**TABLE 3:** Changes from the pre‐test for body composition with the intervention of the exercise trial (Ghana 2022).

Variables	Control (*N* = 95)	Exercise (*N* = 97)	*p*‐value^a^
Weight (kg)			< 0.0001
Pre‐test	76.67 ± 10.80	78.91 ± 13.07	
Post‐test	78.31 ± 10.66	77.19 ± 77.19	
Δ (% Δ)	−1.64 (−2.12)	1.72 (2.20)	
BMI (kg/m^2^)^b^			< 0.0001
Pre‐test	31.19 ± 4.56	31.67 ± 4.31	
Post‐test	31.83 ± 4.45	30.89 ± 4.40	
Δ (% Δ)	−0.64 (−2.03)	0.78 (2.49)	
Muscle mass (kg)			0.033
Pre‐test	25.45 ± 3.62	24.68 ± 2.47	
Post‐test	25.20 ± 3.82	24.89 ± 2.61	
Δ (% Δ)	0.25 (0.99)	−0.21 (−0.84)	
Body fat (%)			< 0.0001
Pre‐test	42.09 ± 5.39	43.19 ± 5.52	
Post‐test	42.32 ± 5.77	41.47 ± 5.91	
Δ (% Δ)	−0.23 (−0.54)	1.72 (4.06)	
Visceral fat (%)			< 0.0001
Pre‐test	10.95 ± 2.04	11.08 ± 1.82	
Post‐test	11.51 ± 2.20	10.72 ± 2.60	
Δ (% Δ)	−0.56 (−4.99)	0.36 (3.30)	
WC (cm)			< 0.0001
Pre‐test	100.03 ± 9.22	102.36 ± 9.57	
Post‐test	102.05 ± 9.33	100.97 ± 10.07	
Δ (% Δ)	−2.02 (−2.00)	1.39 (1.37)	
HC (cm)			< 0.0001
Pre‐test	111.57 ± 8.76	112.87 ± 10.47	
Post‐test	111.78 ± 8.74	110.41 ± 10.86	
Δ (% Δ)	−0.21 (−0.19)	2.46 (2.20)	
WHR^c^			0.130
Pre‐test	0.90 ± 0.07	0.91 ± 0.06	
Post‐test	0.91 ± 0.07	0.92 ± 0.06	
Δ (% Δ)	−0.01 (−1.10)	−0.01 (−1.10)	
WHtR^d^			< 0.0001
Pre‐test	0.64 ± 0.06	0.65 ± 0.06	
Post‐test	0.65 ± 0.06	0.64 ± 0.06	
Δ (% Δ)	−0.01 (−1.55)	0.01 (1.55)	

Abbreviations: BMI, body mass index; HC, hip circumference; N, number; SD, standard deviation; WC, waist circumference; WHR, waist‐to‐hip ratio; WHtR, waist‐to‐height ratio.

^a^ p < 0.05 versus baseline for that group. For within‐group comparisons between post‐test and pre‐test values, a repeated‐measures linear mixed model was used Δ change in means (post‐test – pre‐test).

^b^ Calculated as weight in kilograms divided by height in meters squared.

^c^ Calculated as WC in cm divided by HC in cm.

^d^ Calculated as WC in cm divided by height in cm.

#### 3.2.1 Anthropometrics

After twelve weeks, the intervention group lost a mean of 1.72 kg (a loss of 2.2%) versus a 1.64 kg gain (a gain of 2.1%) in the control group (*p* < 0.0001). Body mass index decreased on average in the exercise group but increased in the control group (+0.78 vs. −0.64 kg/m^2^, *p* < 0.0001). Waist circumference (WC) decreased by a mean of 1.39 cm in the exercise group versus a 2.02 cm increase in controls (*p* < 0.0001). In the exercise group, the mean hip circumference (HC) decreased by 2.46 cm, but the mean increased by 0.21 cm in the controls (*p* < 0.001). A similar trend was observed for WHtR between the exercise group versus the control group (+0.01 vs. −0.01, *p* < 0.0001). However, WHR did not differ between the exercise group and the control groups (−0.01 for both, *p* = 0.130).

#### 3.2.2 Body Composition and Fat Distribution Changes

The exercise group experienced a mean 1.72% decrease in total body fat, which represented a decline from 43.3% to 41.5% versus an insignificant 0.23% mean gain of fat in controls (*p* < 0.0001) (Table 3). A similar trend was observed for visceral fat between the exercise group and the controls (+0.36% vs. −0.56%, *p* = 0.033). Muscle mass however increased by an average of 0.21 kg in the exercise group but decreased by 0.25 kg in the control group with a much less statistical significance compared to body fat and visceral fat (*p* = 0.033 vs. *p* < 0.0001 for both visceral and body fat).

### 3.3 Pre‐Test and Post‐Test Anthropometric and Body Composition Measures Stratified by Baseline BMI (Overweight vs. Obese Participants)

Table 4 presents anthropometric and body composition measures stratified by baseline BMI (participants with overweight vs. participants with obesity).

**TABLE 4:** Pre‐test and post‐test body anthropometric and body composition measures stratified by baseline BMI.

Variables	Baseline BMI < 30 kg/m^2^ (women with overweight)			Baseline BMI ≥ 30 kg/m^2^ (women with obesity)		
	Control (*N* = 46)	Exercise (*N* = 40)	*p*‐value^a^	Control (*N* = 49)	Exercise (*N* = 57)	*p*‐value^a^
Weight (kg)			< 0.0001			< 0.0001
Pre‐test	69.53 ± 7.49	69.50 ± 8.25		83.38 ± 8.92	85.52 ± 11.75	
Post‐test	71.79 ± 7.39	68.33 ± 8.83		84.43 ± 9.61	83.40 ± 12.19	
Δ (% Δ)	−2.26 (−3.20)	1.17 (1.70)		−1.05 (−1.25)	2.12 (2.51)	
BMI (kg/m^2^)^b^			< 0.0001			0.001
Pre‐test	27.55 ± 1.63	27.89 ± 1.22		34.60 ± 3.69	34.33 ± 3.67	
Post‐test	28.67 ± 1.95	27.43 ± 1.50		34.80 ± 4.09	33.32 ± 3.98	
Δ (% Δ)	−1.12 (−3.98)	0.46 (1.66)		−0.2 (−0.58)	1.01 (2.99)	
Body fat (%)			0.001			0.001
Pre‐test	38.46 ± 4.22	39.40 ± 3.60		45.50 ± 3.97	45.85 ± 5.07	
Post‐test	39.03 ± 4.39	37.84 ± 4.32		45.40 ± 5.19	44.01 ± 5.56	
Δ (% Δ)	−0.57 (−1.47)	1.56 (4.04)		0.1 (0.22)	1.84 (4.10)	
WC (cm)			0.025			< 0.0001
Pre‐test	96.08 ± 6.16	95.85 ± 7.03		103.74 ± 10.08	106.92 ± 8.44	
Post‐test	97.84 ± 6.60	95.63 ± 7.10		106.00 ± 9.83	104.72 ± 10.20	
Δ (% Δ)	−1.76 (−1.82)	0.22 (0.23)		−2.26 (−2.16)	2.18 (2.06)	

*Note:* Data are presented as mean ± SD. Δ change in means (post‐test–pre‐test).

Abbreviations: BMI, body mass index; *N*, number; SD, standard deviation; WC, waist circumference.

^a^ *p* < 0.05 versus baseline for that group. For within‐group comparisons between post‐test and pre‐test values, a repeated‐measures linear mixed model was used.

^b^ Calculated as weight in kilograms divided by height in meters squared.

#### 3.3.1 Anthropometry

After twelve (12) weeks, the exercise group with overweight lost a mean of 1.17 kg (a loss of 1.7% vs. 2.26 kg gain, i.e., a gain of 3.2%) in controls (*p* < 0.0001); the exercise group with obesity lost 2.12 kg (a loss 2.5%) versus 1.05‐kg loss (a loss of 1.3%) in the control group (*p* < 0.0001) (Table 4). Body mass index decreased, on average, in the exercise group with overweight (+0.46 kg/m^2^ vs. −1.12 kg/m^2^ in controls, *p* < 0.0001) and the exercise group with obesity (+1.01 kg/m^2^ vs. 0–0.2 kg/m^2^ in controls, *p* = 0.001). Waist circumference decreased by a non‐substantial mean of 0.22 cm (representing 0.2%) in the exercise group with overweight versus 1.76‐cm increase in controls (*p* = 0.025) and decreased by 2.2 cm in the exercise group with obesity versus 2.26‐cm increase in the control group (*p* < 0.0001).

#### 3.3.2 Fat Distribution Changes

The exercise group with overweight had a mean decline of 1.6% in body fat mass, changing from 39.4% to 37.8% versus a mean gain of 0.6% fat in controls (*p* = 0.001). On the other hand, the exercise group with obesity experienced an average 1.8% loss of total fat mass, which represented a decline in the percentage of body fat from 45.9% to 44.0% versus a slight (0.1%) increase in controls (*p* = 0.001) (Table 4).

## 4 Discussion

Given that postmenopausal women are at an increased risk for several health conditions, this current study aimed to test the effectiveness of culturally developed exercise programs on anthropometrics and body composition in postmenopausal women with excess weight gain (overweight and obesity). The results show statistically significant differences between groups in anthropometrics and body composition changes over time and indicate that an exercise program that is culturally designed results in increased health benefits.

Our 12‐week exercise program in postmenopausal women with excess weight gain led to significant decreases in body weight, BMI, WHtR, total body fat, visceral fat, and an increase in muscle mass. Our intervention did not influence WHR; however, there were statistical differences between waist circumference (*p* < 0.0001) and hip circumference (*p* < 0.001) in the intervention group compared with the control group. Therefore, changes in these variables after the intervention might not reflect a change in WHR.

Although the body weight lost at 12 weeks in the exercise group was modest (1.72 kg), a meta‐analysis conducted by Dattilo and Kris‐Etherton [20] and MacMahon et al. [21] revealed that a weight loss of 1 kg decreases triglycerides by 1.9% or 1.5 mg/dL (0.02 mmol/L), serum cholesterol by 1% or 2.3 mg/dL (0.06 mmol/L) and fasting plasma glucose by 3.6 mg/dL (0.2 mmol/L). Thus, a 5‐kg weight loss would result in an 18 mg/dL (1.0 mmol/L) reduction in the average fasting plasma glucose values. Additionally, a study by the Diabetes Prevention Program Research Group [22] found that adults who are overweight with a weight loss aim of 7% and at least 150 min/week of physical activity had a 58% lower risk of type 2 diabetes. Therefore, consistent with the guidelines of the American College of Sports Medicine and the National Heart, Lung, and Blood Institute, a primary weight‐reduction goal should be to lose weight by 5%–10% and maintain this loss over the long term [23].

The major interventional effects between groups were found in total body fat, BMI, WHtR, and muscle mass. The reduction in the intervention group is encouraging as it demonstrates that a decrease in total body fat may lower the chance of developing chronic diseases in postmenopausal women [22, 24, 25], and metabolic syndrome [26]. In the context of weight reduction in adults, the conservation of muscle mass is essential for the maintenance of metabolic capacity, the motor capability of skeletal muscles [27], and the prevention of osteoporosis [28]. The study findings have demonstrated that the preservation of muscle mass is essential. The study found that by using the exercise program, considerable weight reduction may be achieved with an increase in muscle mass.

The women had higher waist circumferences before the intervention in our study. The increase in waist circumference is consistent with other literature findings showing that weight gain in aging mostly occurs around the abdominal region [29, 30, 31]. It is also known that after menopause, visceral adipose tissue accumulation and waist circumference increase above and over the consequences of aging [32]. When compared to the control group in this study, postmenopausal women in the exercise group showed a reduction in waist circumference (1.39 cm vs. −2.02 cm) and hip circumferences (2.46 cm vs. −0.21 cm). An earlier study that investigated the effect of a weight‐management program on the body composition of postmenopausal women with obesity reported weight loss in different areas, namely the thigh and abdominal region [33]. However, the culturally induced exercise program used in the study does not allow differentiation between different fat regions because the “Ampe” in the program involves a total body physical workout.

The analysis within the exercise group stratified by baseline BMI (overweight and obese) demonstrated that the effects of the exercise program were more evident in the body composition (body fat) and anthropometrics (BMI, WC, and body weight) in postmenopausal women with obesity compared with participants who are overweight. For instance, women with obesity experienced a mean 1.84% decline in body fat mass compared with 1.6% in those with obesity. Again, there was a higher reduction in BMI (1.01 kg/m^2^ vs. 0.46 kg/m^2^), WC (2.18 vs. 0.22 cm), and body weight (2.12 kg vs. 1.17 kg) in women with obesity compared with those who are overweight. This variation in effect between postmenopausal women with obesity and overweight should be interpreted carefully because this finding was based on within‐group analyses and could be due to chance. This finding is consistent with a study by Velthuis et al. [34] that investigated 189 sedentary postmenopausal women. Their results revealed that in the exercise group, the effect of the exercise program on the body composition of normal‐weight women was higher compared to women with overweight. Improving the body composition and anthropometrics in postmenopausal women with obesity is relevant; however, there are too few studies to conclude the effects of exercise programs on glucose levels and lipids in postmenopausal women. More studies are needed because elevated blood pressure and lipid profiles are very common conditions in the postmenopausal stage.

Weight reduction interventions are associated with substantially higher drop‐out rates [35]. Velthuis et al. [34] further suggested that adherence might be increased by adding a cognitive behavioral intervention. In this study, the intervention was well accepted by the participants, which may be due to cultural adaptation within the Ghanaian context.

Finally, some limitations should be recognized. The three‐month follow‐up period may be relatively short, especially considering the need for long‐term consistency in exercise programs to assess final results. The step test results and the measurement of anthropometrics alone may have served to encourage the women in the control group to increase their physical activity level, affecting the outcomes of this group. Again, due to logistical issues, only telephonic support was used to improve compliance. In future studies, postmenopausal women's daily dietary intake will be monitored before, during, and after the exercise program.

## 5 Conclusion

This is the first study that investigated the inclusion of cultural recreational physical activity in an exercise program in postmenopausal women with excess weight gain. The study has demonstrated that the intervention program can increase muscle mass, and reduce anthropometric variables (BMI, WHtR, WC, and HC) and body composition (visceral fat, total body fat) in postmenopausal women with an increase in body weight. Weight loss programs that are culturally induced may motivate participants to overcome weight loss challenges [36]. Engagement of culturally adapted exercise programs has important health implications, as shown in this project [37].

## Author Contributions

I.M.B. led the background development, conceptualized the paper, and did the fieldwork and analysis of the data. C.B., A.T.A., and H.M. developed the methodology and were involved in the analysis and proofreading of the paper. I.M.B., C.B. and were involved in structuring, assessing the analysis, and proofreading the paper. All authors were involved in the discussions, conclusions, and recommendations put forward by the paper. All authors further read and approved the final manuscript.

## Ethics Statement

Ethical approval for this study was obtained from the Human Research Ethics Committee at the University of the Witwatersrand, South Africa (Ref no. M190467). In Ghana, approval was also obtained from the Committee on Human Research, Publication and Ethics of Kwame Nkrumah University of Science and Technology, Kumasi (Ref No. 596/19, 621/21). Participation was voluntary and written informed consent was obtained from each participant according to the Helsinki Declaration.

## Conflicts of Interest

The authors declare no conflicts of interest.

## Data Availability

The datasets analyzed in the current study are accessible from the corresponding author on fair request.
